# Allografts: expanding the surgeon’s armamentarium

**DOI:** 10.1007/s10561-022-10015-7

**Published:** 2022-06-28

**Authors:** Norus Ahmed, Volker Eras, Axel Pruß, Carsten Perka, Jan Brune, Tu-Lan Vu-Han

**Affiliations:** 1 German Institute for Cell and Tissue Replacement (DIZG, gemeinnützige GmbH), Haus 42, Köpenicker Str. 325, 12555 Berlin, Germany; 2 Institute for Transfusion Medicine, University Tissue Bank, Charité—Universitätsmedizin Berlin, Charitéplatz 1, 10117 Berlin, Germany; 3 Center for Musculoskeletal Surgery, Charité—Universitätsmedizin Berlin, Charitéplatz 1, 10117 Berlin, Germany

**Keywords:** Bone, Soft tissue, Allograft, Tissue banking, Peracetic acid sterilization

## Abstract

In Germany, bone allografts are widely used and their application in clinics has increased over the years. Successful use of allografts depends on many factors such as the procurement, processing, sterilization and the surgeon’s surgical experience. Tissue banks have provided safe and sterile allografts for decades ranging from hard to soft tissue. Allografts are obtained from various tissues such as bone, tendon, amniotic membrane, meniscus and skin. An advantage of allografts is their wide applicability that has never been limited by indication restrictions thus providing a huge benefit for surgeon’s. The use of the correct allograft in different indications is extremely important. Thereby surgeons have access to various allograft forms such as mineralized, demineralized, freeze-dried, paste, powder, chips strips and putty. The vast options of allografts allow surgeon’s to use allografts in indications they deem fit. Currently, the application of allografts is at the discretion of the expert surgeon. However, regulations are often changed locally or internationally and may impact/limit allograft use to certain indications. Here, we report the different indications where our peracetic acid (PAA) sterilised bone allografts were used as well as general literature on bone allograft use in other indications.

## Introduction

Allografts have become an attractive alternative to autografts, as a result of improved allograft processing methods that have increased their safety and availability. Compared to autografts, the allograft spectrum offers a wide range of allograft forms, providing the surgeon with an abundant supply during surgical procedures, while reducing patient morbidity associated with shorter hospital stays and decreased costs (Vardanian et al. 2009). Thus, the demand for allografts and their clinical applications has increased rapidly in the past 10 years. In Germany alone, bone allograft use in orthopaedic surgery has increased by 74.1% between 2008 and 2018 (Rupp et al. 2021). In turn, autograft use has decreased by 14.3% over the same period (Rupp et al. 2021).

**Fig. 1:**
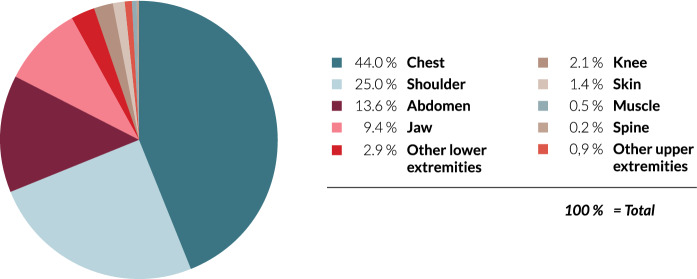
PAA sterilized human acellular dermal matrix use in different locations according to the allograft register. Other lower extremities include hip, ankle joint, tibia/fibula and foot. Other upper extremities include hand, elbow, face and humerus. Data derived from the DIZG allograft database

Allografts vary from hard tissues such as demineralized bone matrix, cancellous and cortical bone to soft tissue allografts like acellular dermal matrix, amniotic membrane, tendons and ligaments. Depending on the surgical field and indication, different grafts can be used (Fig. 1). Grafts are commonly used for the coverage of defects (Lewis et al. 2019) but are also utilized in stabilization (MacDonald et al. 2008) and fusion (Park et al. 2009). Allografts are not limited to certain indications and are applicable in different situations such as wound coverage for burns or necrotizing fasciitis (Henau et al. 2021; Gore and De 2010). The vast uses of allografts provide surgeon’s and patients with multiple treatment options. An advantage of allografts is their wide applicability that has never been limited by indication restrictions thus providing a great benefit for surgeon’s. Over the years, this has allowed allografts to become an extension of the surgeon’s surgical toolbox. The multi-usability of allografts with different indications highlights the importance of such grafts being included in the surgeon’s armamentarium. In our experience, peracetic acid (PAA) sterilized allografts have displayed multi-usability in different indications as reported by surgeon’s. Here, we will report our experiences with PAA sterilised allografts and provide literature reporting on allograft use in different indications.

In Germany, soft and hard tissue allografts are considered as an active pharmaceutical ingredient (Pruß, 2017) but can function as more of a tool under the guidance and expertise of the surgeon. Bone allografts are available in numerous forms such as mineralized, demineralized, freeze-dried, paste, powder, chips strips and putty (Nagi et al. 1997; Schimmel et al. 1998; Schwartz et al. 1996; Slooff et al. 1996; Tägil, 2000). Allografts come from various tissues such as bone, tendon, amniotic membrane, meniscus and skin. They have the ability to affect the microenvironment (Wedel et al. 2017) and provide surgeon’s with the option to customize grafts to satisfy their treatment indications. For the successful treatment of patients with grafts, tissue banks are required to provide safe and sterile allografts.

### Allograft safety and regulations

The safety of allografts is continually improving due to advances in different processing techniques. This improvement is due to a steadily increasing demand for allogenic tissue grafts and the stringent regulations on the removal, processing and storage of such tissues. The basis for this is the EU Directive 2004/23/EC, which was transposed into the German legal system in 2007 as the German law on the Quality and Safety of Human Tissues and Cells Act (Tissue Act) (EU directive, 2006). In addition to this, EU directive and the accompanying tissue law, the German Medicinal Products Act (AMG), the law on organ and tissue donation, removal and transplantation (German Transplantation Act, TPG) as well as the Ordinance on the Manufacture of Medicinal Products and Active Pharmaceutical Ingredients (AMWHV) are all applied. These directives aim to keep allografts safe by detailing the levels of sterility that tissue banks must abide by. The European commission requires a sterility assurance level (SAL) of 10–6 that is currently required for the sterilization procedures (EC, 1990). This SAL is also required by the American Association of Tissue Banks (AATB, 2006). Therefore, diverse sterilization methods are available and implemented to reduce transmission of infectious agents when processing tissues. The type of sterilization is dependent on the tissue bank and grafts can be decontaminated with peracetic acid (Axel Pruss et al. 2003; A. Pruss et al. 2002), gamma irradiation (Nguyen et al. 2007), moist heat (Hofmann et al. 1996) or other methods. However, this is not a prerequisite for viable grafts such as meniscus (Verdonk et al. 2006). Sterilization methods can reduce or alter natural allograft properties. Fideler et al. have reported that dose dependent gamma irradiation can significantly alter the biomechanical features of soft tissue allografts (Fideler et al. 1995). Another example is hydrogen peroxide cleaning that displayed a time dependent significant decrease in osteoinductivity (DePaula et al. 2005). In a previous study, peracetic acid (PAA) sterilization using defatted human spongiosa cuboids displayed a reduction in the titer of viable micro-organisms below the detection limit (A. Pruss et al. 2001; Axel Pruss et al. 2003). Understanding the risks of disease transmission via allografts has been investigated and PAA-sterilized allogeneic musculoskeletal tissue grafts were considered as safe (Brune 2019). PAA sterilization, a validated method that eliminated micro-organisms from PAA-treated bone transplants (A. Pruss et al. 2001) is utilized by the Deutsches Institut für Zell- und Gewebeersatz (DIZG) tissue bank.

In order to continually improve the allograft spectrum and safety, in addition to the sterilization technique, we have implemented a database on allograft use. Information on graft use in different indications has been collected by the DIZG in the form of an allograft register (Perka, 2021) for the past 12 years. The data collected is based on the use of PAA sterilized allogeneic hard and soft tissue transplants. This data represents a long historical range (2009 to present) of allograft use and leads to the scientific evaluation and development of special patient treatment options for bone and soft tissue defects. The register allows for collected data to be converted into an evaluable form in cooperation with the German Society for Orthopaedics and Trauma Surgery (Perka, 2021). Understanding the different indications that allografts have been successfully applied in permits for constant improvements and advancements for patient care.

### Bone allografts

Bone allografts have successfully been applied in osseous defects that constitute different microenvironments such as traumatic (e.g. fractures), degenerative (e.g. arthritis), neoplastic (e.g. malign and benign tumors), hypoplastic (pseudarthrosis), peri-implant, as well as for the purpose of bone fusion (e.g. arthrodesis, spinal fusion) (Pastl & Schimetta, 2021)(R. M. Wilkins & Kelly, 2003). With the relative abundance of available allografts, the surgeon is able to overcome larger osseous defect sizes, in contrast to autografts (Temple and Malinin, 2008). Thus, allografts display impressive multi-usability, which can be further expanded by additional intraoperative processing and pairing of allografts with active agents such as antibiotics or patient’s bone marrow.

## Demineralized bone matrix (DBM)

Demineralized Bone Matrix (DBM) is a bone graft derived from human allograft bone. DBM undergoes acidic processing to remove the mineral components of the bone whilst leaving the extracellular matrix made up of collagen and non-collagenous proteins.

### DBM use in spinal fusion

Spinal surgery has utilized DBM, a recent publication by Balling et al. compared the use of PAA sterilized DBM putty to traditional autologous iliac crest bone grafting for fracture treatment with thoracolumbar anterior single-level interbody fusion in a patient cohort of 30 individuals. Biological fusion, measured in Hounsfield units (HU) by CT scans, occurred in 80% of the DBM group and in 57% of autologous iliac crest bone grafting after 9 months (Balling and Weckbach, 2020). Park et al. conducted a clinical trial study with 31 patients with single- to multilevel cervical disk disease and surgical indication for anterior cervical fusion, and investigated the fusion rate when pairing polyether ether ketone (PEEK) cages with DBM mixed with autologous bone chips (Park et al. 2009). After 12 months, a fusion rate of 97% was recorded displaying successful treatment (Park et al. 2009). Vaccaro et al. (2007) investigated DBM use in posterolateral lumbosacral spinal fusion for 73 patients diagnosed with degenerative disk disease or degenerative spondylolisthesis. Patients were treated with either DBM putty enriched with aspirated bone marrow, DBM putty with iliac crest autograft or autograft. After 24 months, fusion rates were 63% for DBM/bone marrow, 70% in the DBM autograft group and 67% in the autograft group. This data suggests that DBM provides similar results to that of autografts in posterolateral spinal fusion (Vaccaro et al. 2007).

### DBM use in fractures

Demineralized bone matrix use in long bone fractures has been documented in a level II (Lindsey et al. 2006) and two level III (Bibbo & Patel, 2006; Cheung et al. 2003) comparative studies. DBM was combined with bone marrow aspirate in diaphyseal long bone fractures in a level II prospective randomized pilot study and in a level III retrospective study, DBM mixed with cancellous chips for treatment of periarticular fractures. The second level III study combined DBM with calcium sulfate and vancomycin for treating displaced intra-articular calcaneal fractures. Successful use of DBM in these studies has provided an alternative to autologous grafting for such fractures. These examples highlight that the combination of allografts with active agents can increase their purpose.

### DBM use in long bone

In the literature, tibial defects were also treated with DBM as described by Hatzokos et al. (2011). It was stated that DBM application with autologous bone marrow concentrate provides an equivalent and safe alternative to that of autologous bone grafting in the effective management of docking sites during distraction osteogenesis. A level IV prospective clinical study (Wilkins et al. 1999) compared the use of calcium sulfate pellets mixed with DBM. Wilkins et al. (1999) reported DBM use for patients undergoing bone grafting for benign bone lesions, non-union of long bones and osteomyelitis. The DBM mixture provided a safe treatment for bone regeneration with no graft related complications.

### DBM use in ankle and foot fusion

Thordarson and Kuehn, (2003) described a level III study comparing different DBM use in complex ankle or hindfoot fusion. In this study, 63 patients undergoing complex ankle or hindfoot fusion were treated with DBM at the fusion site. The authors aimed to stimulate fusion whilst comparing two different types of DBM. A putty form of DBM and crushed cancellous bone allograft were used in 37 and 26 patients, respectively. Non-union occurred in five (14%) patients and two patients (8%) in the putty and crushed cancellous bone groups, respectively. Non-union rates for ankle and hindfoot fusions were approximately 10% and both the commercially available DBMs displayed no significant difference between their union rates (Thordarson and Kuehn, 2003).

### DBM use in maxillofacial surgery

A retrospective study by Kuhls et al. investigated the treatment of cystic mandibular defects with PAA sterilized DBM (n = 50) compared to patients without DBM (n = 40) (Kuhls et al. 2001). Indeed, patients treated with DBM showed faster bone regeneration compared to those without DBM.

### DBM use in non-unions

Studies investigating the application of DBM in the treatment of non – unions have shown successful outcomes and included level III (Hierholzer et al. 2006) and one level IV study (Wilkins et al. 2003). Hierholzer et al. reported the use of DBM in non-union or delayed union of humeral shaft fractures and showed no significant differences compared to autologous iliac crest bone graft (Hierholzer et al. 2006). Wilkins et al. used a percutaneous approach in the application of DBM combined with autologous bone marrow and presented successful treatment in long bone fractures with outcomes comparable to iliac crest autologous bone grafting (R. M. Wilkins et al. 2003).

### DBM use in bone cysts

DBM can be used in the treatment of bone cysts and different studies have shown good results leading to high healing rates with minimal complications. A retrospective comparative study conducted by Di Bella et al. (2010), showed that unicameral bone cysts treated with a single injection composed of DBM and bone marrow concentrate achieved higher healing rates without fracture complications compared to the standard percutaneous corticosteroid injections. Successful use of DBM has been documented by others (Rougraff and Kling, 2002) and for the treatment of active unicameral bone cysts in children (Kanellopoulos et al. 2005).

### DBM use in tumor surgery

A retrospective clinical study conducted by Wilkins et al. investigated the use of DBM in the treatment of non-unions in various bones after surgical removal of benign tumors (R. M. Wilkins and Kelly, 2003). The authors concluded that DBM putty is suitable as a bone void filler in bone defects after tumor surgery.

### DBM use in osteonecrosis of femoral head

Other reported uses of DBM include a level III study in the treatment of large osteonecrosis lesions of the femoral head. Feng et al. (Feng et al. 2011) retrospectively compared patients undergoing free vascularized fibular grafting and DBM with autologous cancellous bone grafting. Femoral head osteonecrosis was treated successfully with DBM leading to an improvement in the mean Harris hip score.

### DBM use in acetabular revision

A level IV retrospective study by Etienne et al. investigated the use of allograft cancellous bone chips mixed with DBM for the filling of the cavitary defects in 20 patients who had cementless acetabular revision arthroplasty. After a 2-year follow-up, the DBM mixture was fully incorporated in 18 patients. The authors reported that the DBM mixture provided acceptable clinical and radiographic results for osteolytic acetabular defect treatment (Etienne et al. 2004).

### DBM use in shoulder instability

The use of DBM has also been reported in the treatment of shoulder instability. A published technical note by Moroder et al. displayed the use of an allogeneic demineralized spongy bone matrix for the treatment of anterior shoulder instability with small to intermediate glenoid defects using the arthroscopic Bankart-Plus procedure (Moroder et al. 2018). The authors report that the insertion of the allograft increases the volume of the generated capsulolabral bump that is more beneficial than the conventional Bankart repair. This newly reported indication for DBM further expands the potential uses of allografts, leading to greater potential for patient treatment. Clinicans are continually improving their treatment methods for patients and providing such allografts in these indications allows for continuous advancement. An ongoing study using PAA sterilized demineralized bone allograft is also being investigated in the arthroscopic Bankart-Plus procedure (unpublished data).

## Cancellous bone

Cancellous bone is spongy and known as trabecular bone, it is highly porous and made of hard and soft tissue components found at the epiphyses of long bones and in vertebral bodies. Trabecular bone is a honeycomb-like network and is comprised of many open spaces that are connected by interconnected rods and plates known as trabeculae. The trabeculae are interspersed within the bone marrow compartment and arranged to optimize load transfer (Bayraktar et al. 2004; Oftadeh et al. 2015).

### Cancellous bone use in tunnel filling

Cancellous bone allograft has been described in a wide range of settings and has been recently used for patients with recurrent instability after anterior cruciate ligament (ACL) reconstruction (Kodach et al. 2008). A recent study by Prall et al. investigated the use of PAA treated freeze-dried cancellous bone chips and compared its use with autologous cancellous bone grafting (Prall et al. 2020). In the study, it is reported that cancellous bone allograft was used for filling of enlarged or misplaced tunnels in two-staged revision ACL surgery. Filling rates were comparable to that of autologous bone grafting. Cancellous allograft success in such indications provide suitable alternatives to autologous grafts in such procedures including their unrestricted quantity and lack of donor site morbidity.

### Cancellous bone use in bone cysts

Peracetic acid sterilized allogenic cancellous bone chips have also been used in the treatment of unicameral bone cysts (Toepfer et al. 2016). Toepfer et al. concluded that allogenic cancellous bone are favorable due to their advantage in terms of osteointegration (Toepfer et al. 2016).

### Cancellous bone use in fractures

Rajan et al. conducted a prospective, randomized trial investigating the use of cancellous chips for the repair of comminuted distal radius fractures (Rajan et al. 2006). The authors noted no adverse effects were detected using allografts for the treatment of radius fractures and cancellous allografts provide an alternative to autografts (Rajan et al. 2006). Autografts also lead to complications as shown by (Aurich and Hofmann, (2020) who reported the application of PAA cancellous bone allograft to fix autograft harvesting complications. (Aurich and Hofmann, (2020) published a case report demonstrating the use of cancellous bone allograft to fill the defect of the bone graft harvest site during the treatment of a displaced avulsion fracture of the anterior superior iliac spine (ASIS) following iliac crest bone harvesting. Martanto et al. compared the use of bone autograft and compared it to freeze-dried bone chip allograft in small defects of long bones. The authors reported no significant difference between both groups in bone healing (Martanto et al. 2020).

### Cancellous bone use in spinal surgery

Patients with degenerative spinal disease were treated with monosegmental spondylodeses. PAA sterilized human allogenic cancellous bone allograft was used. After 12 months, allogenic cancellous bone displayed similar fusion rates to that of autogenous iliac crest cancellous bone for cage filling (Putzier et al. 2009). These allografts all provide surgeon’s with ability to use graft types that best suit the patient’s need whilst still providing equivalent clinical outcomes to that of autologous grafts.

### Cancellous bone use in impaction bone grafting

Impaction bone grafting techniques are often used when the surgeon encounters large cavitary acetabular defects. This technique is used for the treatment of large ectatic femoral metaphysis or diaphysis by packing defects with compressed particulate graft (Oakes and Cabanela, 2006). (Patil et al. (2012) investigated the use of cancellous bone in the reconstruction of major acetabular bone defects. Impaction bone grafting for acetabular reconstruction was reviewed from 168 total hip arthroplasty from 1997 to 2008. The authors compared autograft, cancellous allograft and DBM for bone defect filling. Radiographic analysis displayed that impaction of grafted bone incorporated well for allograft and autograft leading to restored bone stock (Patil et al., 2012). In another study, seven patient cases of acetabular revision were treated with impacted freeze-dried cancellous bone chips. The authors reported that radiographic analysis of freeze dried cancellous bone chips is acceptable in acetabular reconstructions (Thien et al. 2001). The average follow-up ranged from 5 to 9 years and the survival rate was 86%.

### Cancellous bone use in osteotomy

Santic et al. reported cancellous bone allograft use in 310 knees in 284 patients between 2000 and 2005 for medial opening wedge high tibial osteotomy (MOWHTO)(Santic et al. 2010). The follow-up of this study ranged from 3 to 8 years with an average of 5.9 years. Cancellous bone allografts were implanted and healing occurred in 90% of cases within 12 weeks. The authors regard cancellous bone allograft as a satisfactory choice in bone healing for medial opening wedge high tibial osteotomy (Santic et al. 2010). Cancellous allograft use for osteotomy has been described by Cho et al. in another study investigating MOWHTO comparing both autogenous bone graft and cancellous bone allograft (Cho et al. 2013). In this study, 51 patients (52 knees) dating from 2007 to 2010 were divided into 2 groups. Group 1 (n = 29) received allograft while group 2 (n = 23) were treated with allogenic cancellous bone chips. Radiographic measurements displayed no significant difference between the groups with an average union rate of 11.7 weeks and 12.1 weeks for group 1 and 2, respectively. The authors conclude that cancellous bone can be used as an alternative to autologous bone in medial open wedge high tibial osteotomy (Cho et al. 2013).

### Cancellous bone use in partial meniscal repair

PAA sterilized allografts have also been used by surgeon’s in rarer procedures according to the DIZG allograft register. An ongoing study with a clinical follow up is investigating the use of cancellous bone in partial meniscus repair (unpublished data).

## Cortical bone

Cortical bone is a dense structure and is the outer bone surface that protects the internal cavity. As part of the microstructure, cortical bone contains osteons that are produced in the remodelling process. A central vascular canal is contained in osteons and is known as the Haversian canal that is surrounded by lamellae (Bernhard et al. 2013). Cortical bone has also been used in various indications and surgeries and provides a vast array of options for patient treatment. PAA sterilized cortical bone can be used in the form of a fine-threaded screw that combines the benefit of a human bone matrix (Shark Screw®). The Shark Screw® has been used in different locations such as osteosynthesis in hand, elbow, knee, and foot surgery.

### Cortical bone use in hand and foot surgery

Pastl and Schimetta, (2021) reported the use of the cortical bone screw graft in hand surgeries and foot surgery. This data was reported from a single surgeon case series of 32 patients with an average follow-up time of 1 year. The authors note high patient satisfaction with low post-operative pain levels, complication rates and a 100% fusion rate (Pastl and Schimetta, 2021). The PAA sterilized cortical human bone screw (Shark Screw®) has also been described in a 52-year-old patient with hallux rigidus (Brcic et al. 2021). The patient underwent arthrodesis of the first metatarsophalangeal joint using Shark Screw®. Histological analysis displayed a vascularized graft that resulted in bone healing. The authors suggest that the PAA sterilized bone screw provides fast bone healing with no immunological rejection. The authors concluded that allogenic cortical bone is safe and has excellent graft incorporation into host bone (Brcic et al. 2021). Additionally, the use of human bone screws provides many advantages for surgeon’s and patients. Human bone screws do not have to be removed as in the case for metallic screws, reducing the need for a secondary surgery. The high safety standards of allograft processing by tissue banks prevents the risk of immune rejection and complications that occur when using traditional metallic screws.

### Cortical bone use in maxillofacial surgery

Wallowy et al. reported the use of cortical bone in lateral ridge augmentation of the maxilla and mandible in patients with alveolar ridge atrophy (Wallowy and Dorow, 2012). In this study, the authors reported the use of different forms of cortical bone allograft. The authors combined both a PAA sterilized flexible thinned allogeneic cortical graft with PAA sterilized particulate allogeneic cortical bone with a high clinical success (Wallowy and Dorow, 2012). In this case, the ability to combine different types of allografts demonstrated effective combination treatment.

### Cortical bone use in osseous defects

Benign bone tumors often leave a defect in the bone after tumor removal. The use of cortical bone allograft for metaphyseal and metadiaphyseal surgical bone defects has been reported with high clinical success in 97 patients (Temple and Malinin, 2008). Microparticulate cortical bone allograft was used for packing of osseous defects and provided an effective alternative to autogenous bone graft (Temple and Malinin, 2008). Houdek et al. investigated the use of large cortical allografts with 18 pediatric patients undergoing lower extremity limb salvage with massive cortical bone allograft and free fibular transfer using the Capanna technique (Houdek et al. 2016). The patients consisted of 9 boys and 9 girls ranging from 5 to 18 years old with an average age of 11 years old. The follow-up period ranged from 2 to 15 years with an average follow-up time of 8 years. A 94% limb salvage rate was observed with the use of massive cortical bone allograft and provided a reliable method for large bony tumor reconstruction in the lower extremity (Houdek et al. 2016).

### Cortical bone use in non-union

Cortical struts are useful in the treatment of shaft non-union. A retrospective study reviewed the use of cortical allograft struts in the treatment of humeral non-union with 57 patients at the Rizzoli Orthopedic Institute (Marinelli et al. 2009). The authors reported that in their experience, cortical allograft struts provide a standardized, reproducible technique with low complication rates for the treatment of severe atrophic non-union. Hornicek et al. (2001) also reported the use of cortical allografts in the form of freeze- dried tibial and femoral plates and fibular shafts for diaphyseal humeral non-unions. All humeral non-union had united in this study.

## Conclusion

This paper describes different bone allografts and the literature regarding their successful applications in yet underexplored surgical indications, for example in rare surgical cases. Allograft studies have demonstrated successful osteointegration in different microenvironments (e.g. infectious, traumatic, degenerative, tumorous) and surgical sites. The paper also highlights the indications where our PAA sterilised allografts have been used and the different aspects of allograft processing, qualities and active properties are summarized. These sterile and safe allografts provide different options ranging from hard to soft tissues. These types of allografts equip clinicans with a natural toolbox for the treatment of patients. Depending on the surgical sites these grafts are available in multiple forms and range from powder to bone struts. Allografts not only give clinicans an array of materials that can be utilized but also display the ability to treat different indications whilst still allowing for combinations with other allografts or solutions to transfer specific proporties to the supporting material. In addition, allografts come at a less limited supply, reduced morbidity, shorter hospital stays, decreased costs and are readily available in various sizes and shapes compared to their autograft counterparts. The use of safe sterilized allografts in various indications is of great benefit to not only the surgeon’s but the patients too. Allograft use in surgeries continue to evolve and the use of allografts is increasing over time, thus maintaining a vast catalogue of allografts for different treatment options, while ensuring allograft safety and quality, is of great importance. Currently allograft variability and multi-usability is maintained with the surgical indication residing with the operating surgeon, thus leaving the potential for innovative and novel applications in the hands of these professionals. As professionals, surgeon’s should be able to decide on the appropriate allograft to use in the specific situations. Overall, the use of a register, the collection of patient follow-ups and clinical outcome data is essential in improving the evidence base. Additionally, the safety and sterility of allograft processing aids in reducing the risk of infective agents and does not lead to a clinically significant immune reaction of the patient. In turn, providers of sterile allografts take on the role of ensuring the safety of the material. From our view, allograft use seems to be expanding in the hands of surgeon’s. Limiting such use may in fact hinder patient care and satisfaction in indications where different allografts can be used. Therefore, considering allograft safety, our experiences and the vast literature describing their use in various indications only strengthens the importance of allografts and their role as an integral part of the clinicans' armamentarium. Allograft use in different settings as reported in this paper exhibits the importance of allografts in human health and recovery.

## Data Availability

Not applicable.

## Abbreviations

ACL: Anterior cruciate ligament
HADM: Human acellular dermal matrix
HU: Hounsfield Units
MOWHTO: Medial opening wedge high tibial osteotomy
PAA: Peracetic acid
PEEK: Polyetheretherketone
